# Development of a Color-Changing Face Mask for Fever Detection Applications

**DOI:** 10.3390/ma18092042

**Published:** 2025-04-29

**Authors:** Nareerut Jariyapunya, Sunee Hathaiwaseewong, Nanjaporn Roungpaisan, Mohanapriya Venkataraman

**Affiliations:** 1 Department of Textile Engineering, Faculty of Engineering, Rajamangala University of Technology Thanyaburi, Pathum Thani 12110, Thailand; nanjaporn_r@rmutt.ac.th; 2 Department of Material Engineering, Faculty of Textile Engineering, Technical University of Liberec, 461 17 Liberec, Czech Republic; mohanapriya.venkataraman@tul.cz

**Keywords:** thermochromic, face mask, color-changing, thermal conductivity, fever

## Abstract

This study focused on developing a color-changing fabric face mask for fever detection. Reversible Thermochromic Leuco dye (RTL) was applied as an indicator to alert wearers of elevated body temperatures, with the color change occurring at 37.5 °C. Five fabric types Polyethylene (PE), cotton (CO), a cotton–polyester blend (TC), polyester (PL), and Polyamide (PA) were coated with blue RTL to evaluate their color change responsiveness. The results showed that fabrics with higher thermal conductivity (***λ***), thermal absorptivity (***b***), and heat flow (***q***) exhibited faster color transitions. RTL-coated PE fabric demonstrated the best performance, with a thermal absorptivity of 312.8 Ws^0.5^m^−2^K^−1^ and a heat flow of 2.11 Wm^−2^, leading to a rapid color-change time of approximately 4.20 s. Although PE fabric had a lower thermal conductivity (57.6 × 10^−3^ Wm^−1^K^−1^) compared to PA fabric 84.56 (10^−3^ Wm^−1^K^−1^), the highest thickness 0.65 mm of PA fabric slowed its color-change reaction to 11.8 s. When selecting fabrics for optimal heat transfer, relying solely on fiber type or thermal conductivity (***λ***) is insufficient. The fabric’s structural properties, particularly thickness, significantly impact thermal resistance (***γ***). Experimental results suggest that thermal absorptivity and heat flow are more effective criteria for fabric selection, as they directly correlate with color-change performance.

## 1. Introduction

Disposable face masks, which are made from non-biodegradable materials such as various types of plastics, have rapidly become a significant waste problem during the pandemic [1,2,3]. This is especially true as people worldwide rely on masks daily to prevent the spread of disease [4]. The accumulation of waste from single-use masks not only increases the volume of waste in the environment but also poses threats to wildlife and ecosystems when these masks end up in water bodies or poorly managed landfills [5,6]. One viable solution to mitigate this issue is to shift to reusable masks, which are made from durable materials that can be washed and reused multiple times [7,8]. Moreover, the reusable masks or close-fitting masks demonstrated a protection efficiency of over 90%, comparable to that of certified industrial masks and disposable masks [9] and against the particulate matter of size ranging from 0.01 to 0.3 µm [10]. Switching to this type of mask can significantly reduce the amount of waste generated by disposable masks [11]. Fabric face masks, which have gained significant popularity as a reusable alternative to disposable masks, have undergone considerable development to improve their functionality and user comfort [12,13,14]. Initially, simple flat fabric masks with pleats on the sides were the standard design, offering basic protection and ease of production. However, as more people adopted reusable masks in their daily lives, feedback regarding fit, comfort, and breathability led to innovations in mask design. One of the most notable advancements is the introduction of 3D Fabric face masks [15].

Reversible Thermochromic (RT) materials are intelligent materials that change color in response to temperature variations, exhibiting either reversible or irreversible behavior during heating and cooling cycles. The Reversible Thermochromic Leuco dye (RTL) is a dye that can switch between two states, one colored and the other colorless, with the transition occurring either gradually or abruptly [16,17,18]. The mechanism of the RTL system operates based on the interplay of two main interactions. The first interaction occurs between the leuco dye and the color developer through an acid–base reaction. At lower temperatures, the dye and the color developer interact strongly, often forming a protonated (colored) state, which is responsible for the visible color in the lower-temperature phase. The second interaction takes place between the color developer and the solvent via non-covalent forces. As the temperature rises, the solvent melts, disrupting the acid–base interaction between the leuco dye and the color developer. This disruption causes the dye to adopt its deprotonated (colorless) form, leading to the disappearance of the visible color [16,18,19,20,21]. The melting point of the solvent is a crucial parameter in determining the temperature at which the thermochromic effect occurs. By carefully selecting solvents and dye–developer compositions, the transition temperature of the thermochromic material can be tuned for specific applications [22].

The potential application involves incorporating RTL color-changing technology that responds to temperature variations in fabric mask indicator applications. The first consideration is designing the face mask to closely contact the skin, allowing heat transfer from the skin to the fabric-coated RTL layer. The second critical factor supporting RTL color change is the fabric’s ability to absorb heat from the skin, particularly in the cheek area, to activate the RTL and induce a color change [23,24]. Thermal conductivity (***λ***) and thermal absorptivity (***b***) are intrinsic material properties that determine the ability to transfer or absorb heat. These factors influence the accuracy of RTL color changes in real time when the melting point is set at 37.5 °C on the fabric. The normal core human body temperature is approximately 37 °C, fluctuating by around 0.5 °C throughout the day [25,26]. In the case of a fever, the core body temperature often increases by more than 0.5 °C [25]. Fever classification commonly defines a lower threshold of 37.3–38 °C as the onset of fever or low-grade fever [25,27].

It was found that the thermal conductivity of fibers varies, affecting the heat transfer properties of textile materials [28,29]. The use of different types of fibers with distinct thermal conductivity or heat transfer characteristics means that the fidelity of heat detection in RTL-coated fabrics when undergoing color changes at predetermined temperature points may vary. This also helps to circulate heat and keeps overheating in check, leading to irritation and unpleasant experiences while in use. Improved identification of breathable fabrics, along with the accurate measurement of heat distribution and overall comfort for each type.

The integration of color-changing RTL masks enables rapid, non-invasive fever screening, offering significant public health benefits. These masks provide a visual alert when a wearer’s temperature exceeds 37.5 °C, aiding early detection in high-risk settings. Unlike traditional sensor-based or smart textile systems that require power and electronics, this RTL mask is a passive, non-electronic solution that changes from colored to colorless upon fever detection.

The novelty of this work lies in its use of thermochromic materials on water-repellent fabric, enabling faster and more reliable heat transfer and color response. Compared to existing fever-detecting masks, this design is more accessible, cost effective, and user-friendly, requiring no digital components or infrastructure. Additionally, the mask is washable, making it suitable for repeated use and enhancing its practicality for everyday applications. The primary aim of this study is to investigate fabric types capable of efficiently transferring facial heat to the RTL layer to ensure a responsive and visible color change. This knowledge will be applied to enhance the effectiveness of color-changing fabric masks for reliable fever detection on the facial surface. The inclusion of color-changing RTL masks for screening individuals with high body temperature provides considerable public health benefits to allow rapid and non-invasive identification of fevers. These masks trigger a physiological alert by indicating visually when a wearer has a temperature higher than 37.5 °C, making it easier to identify potentially sick individuals in crowded or high-risk environments. Early detection can help to control infectious disease spread via timely intervention. Most prior work on fever-detecting face masks has been limited to embedding thermal sensors or smart textiles to detect temperature variations. But these methods tend, by necessity, to involve external power supplies, complex electronics, and digital readouts, all of which may not be feasible to implement widely. A new type of color-changing RTL mask is being developed as an alternative non-electronic solution for immediate temperature detection of a visual change. When the body temperature of the wearer reaches 37.5 °C or higher, this mask indicates an alert by changing from the colored state to the colorless state, so such a type of mask can be recognized as an alarm because it has an immediate intuitive concept. This research focuses on selecting fabric types that efficiently conduct heat from the skin to the RTL fabric layer, ensuring an effective and responsive color change.

## 2. Materials and Methods

### 2.1. Materials

This study examines the selection of fabrics for coating with Reversible Thermochromic Leuco dye (RTL) and evaluates their sensitivity to color change at RTL’s melting point. Fabrics with different fiber compositions were chosen to investigate their heat absorption properties, based on the hypothesis that fiber type influences heat transfer and accelerates color change. A total of five fabric types were tested, as shown in Table 1.

### 2.2. Methodology

#### 2.2.1. RTL Mechanism and the Fabrics Coated with RTL Process

RTL microcapsule is used to change color in response to temperature variations and a reversible thermochromic blue color was set the melting point. When the thermochromic material is coated by screen printing technique onto fabric, it induces a color change through a heat-induced chemical structural transformation. A blue-colored reversible thermochromic with an activation temperature higher, which becomes colorless upon reaching the activation temperature. The color change is reversible when the temperature drops below melting point.

**Figure 1:**
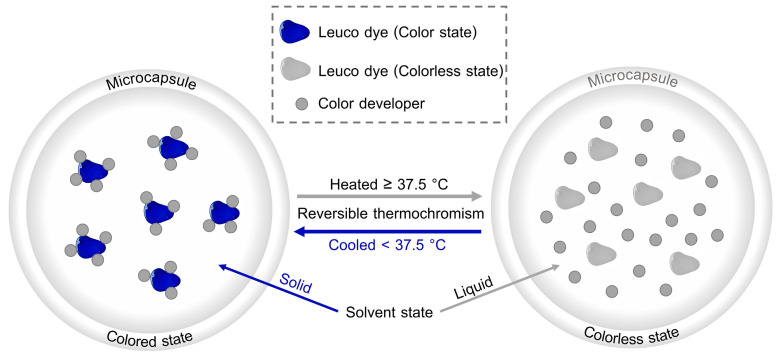
The mechanism of RTL microcapsules.

As illustrated in Figure 1, the mechanism of RTL microcapsules in this study is based on reversible color changes induced by temperature variations around the melting point of 37.5 °C. The primary components involved in this process are RTL and the color developer. When the temperature exceeds 37.5 °C, corresponding to the melting point of the solvent in its liquid state, RTL undergoes deprotonation, resulting in a transition to a colorless state. Conversely, when the temperature falls below 37.5 °C, the solvent crystallizes into a solid state, allowing RTL to regain its protonated form and transition into a blue-colored state.

**Figure 2:**
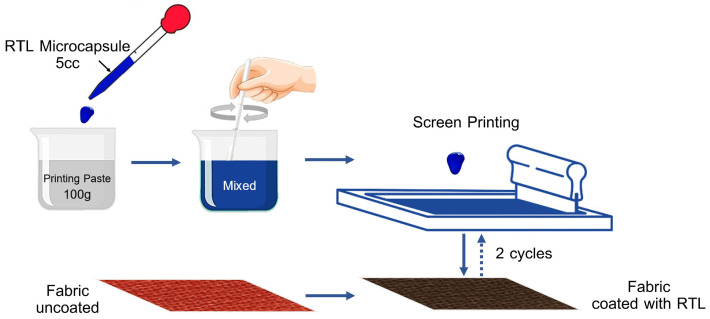
Flow chart of thermochromic coated fabric by the screen printing process.

Figure 2 presents the flowchart depicting the application of thermochromic coating onto fabric via the screen printing method, utilizing a ratio of 5 cc of thermochromic pigment to 100 g of pigment paste. The blue RTL was designed with a phase transition temperature of 37.5 °C. This microcapsule exhibited specific properties, including a pH range of 0.5–0.7, a specific gravity of 0.9–1.2, a non-volatile matter content of 31–35%, and an average particle size of 1–5 µm. The printing paste formulation employed in the process is detailed in Table 2 and the physical properties of coated fabrics are shown in Table 3.

#### Antibacterial and Water-Repellent Treatment on the Shell Fabric

**Figure 3:**
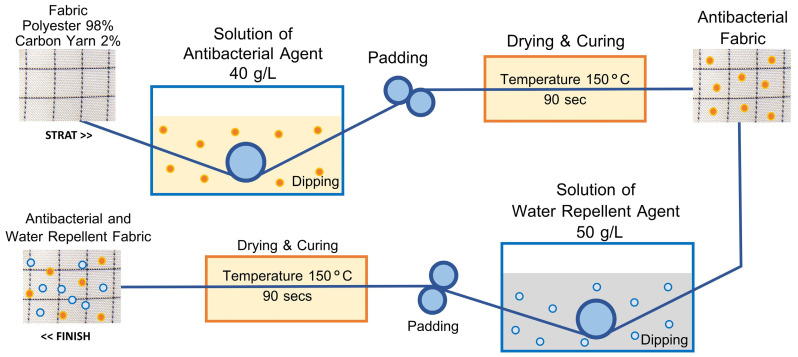
Treatment process for antibacterial and water-repellent of shell fabric.

The shell fabric receives antibacterial and water-repellent treatments since it acts as the initial barrier against environmental contaminants and moisture with pathogens. The antibacterial treatment reduces the risk of contamination by blocking microbial growth on the mask’s outside surface. The water-repellent treatment blocks liquid entry to preserve mask dryness and its protective function. If these treatments are applied to the mask’s inner layer they might produce skin irritation while decreasing breathability which would lead to reduced wearer comfort. The mask retains its protective qualities while remaining comfortable and safe throughout prolonged use by concentrating on the shell fabric. This step focuses on developing the properties of the shell fabric by selecting finely woven plain weave fabrics made of 98% polyester microfiber blended with 2% micro carbon fibers. The fabric is then treated to enhance antibacterial and water-repellent properties, as illustrated in Figure 3.

#### Design and Development of the 3D Face Mask

**Figure 4:**
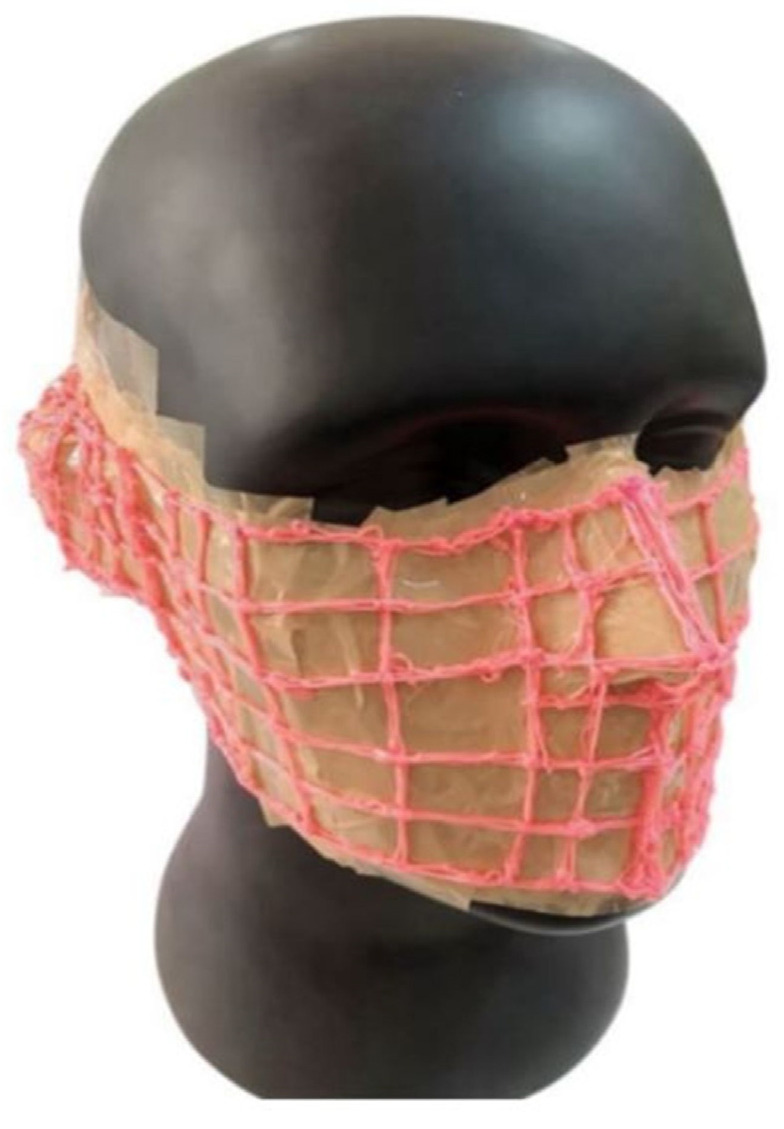
Pattern drafting technique for face masks.

The fabric mask design was developed using the draping technique on a human facial mannequin. Liquid polymer lines were applied to assist in pattern drafting, ensuring a precise and comfortable fit for the mask, as illustrated in Figure 4.

**Figure 5:**
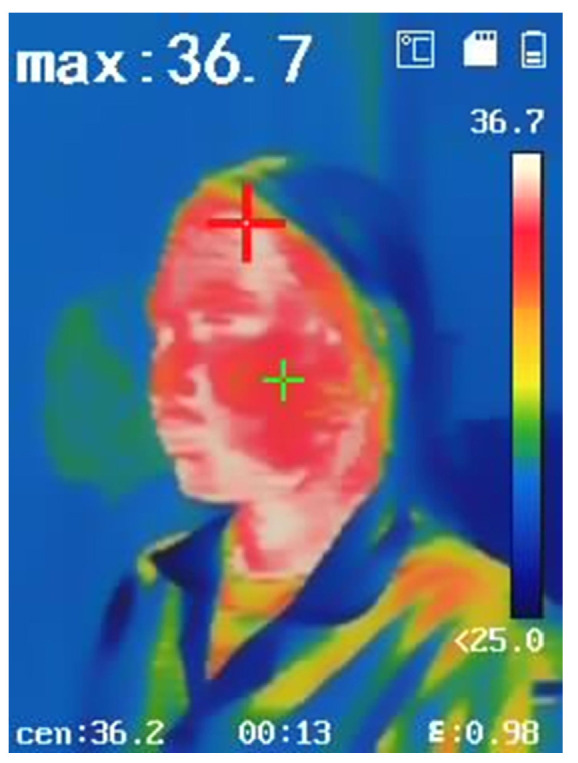
Thermal imaging of the facial region.

The analysis of facial areas susceptible to heat buildup, especially where the mask makes direct contact with the skin, was performed using a thermal imaging camera Hikvision DS-2TP31B-3AUF (Hangzhou Hikvision Digital Technology Co., Ltd., Hangzhou, China), as shown in Figure 5. The thermal image presents a gradient color map, identifying regions of the face where the mask fits tightly without air gaps. The image reveals that the areas around the cheeks and near the ears, indicated by red coloration, exhibit high heat accumulation. This information can be used to optimize the design of the facemask and refine the pattern construction of the RTL fabric to improve its heat absorption effectiveness.

**Figure 6:**
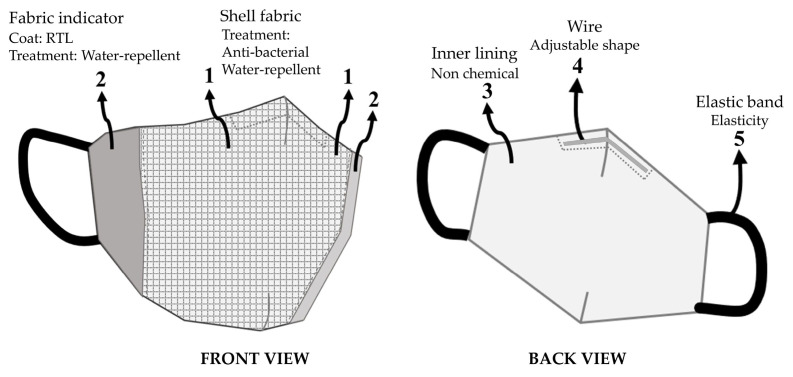
The design of a color-changing face mask (1—shell fabric of facemask, 2—coated RTL for color changing, 3—inner lining, 4—wire, and 5—elastic band).

The design incorporated thermochromic coated on fabric sections strategically placed to react and change color when exposed to temperatures above 37.5 °C, providing a functional and visually responsive feature. A draft of the concept of color-changing fabric for fever measurement applications is shown in Figure 6.

In Figure 6, No. 1 refers to a shell fabric made from woven plain weave material composed of 98% polyester microfiber blended with 2% micro carbon fibers. This fabric has a yarn density of 37 threads per cm for the weft and 50 threads per cm for the warp, a weight of 93.90 GSM, and a thickness of 0.16 mm. Additionally, the micro carbon fibers help reduce static electricity, minimizing the adhesion of droplets from coughing or sneezing to the fabric or mask during use. Furthermore, the fabric has been treated to enhance its antibacterial and water-repellent properties, improving the functionality of the fabric face mask.

No. 2 is coated with a blue RTL. The color change sensitivity will be determined in the upcoming testing stage. The design of this fabric mask ensures that the part in direct contact with the face effectively transfers heat to the fabric coated with RTL. Moreover, after coating with RTL, the fabric was treated to enhance its water-repellent properties, providing protection against moisture and water.

No. 3 refers to the inner lining of the fabric mask, made from 100% cotton with a plain weave structure. This fabric has a yarn density of 42 threads per cm for the weft and 55 threads per cm for the warp, a weight of 78.65 GSM, a thickness of 0.14 mm, and is free from any chemical treatments to minimize irritation during wear.

No. 4 is a wire inserted along the nose area to adjust the mask for a snug fit, reducing gaps while wearing the fabric mask.

Finally, No. 5 is an elastic band used to secure the mask around the ears, ensuring a proper fit during use.

#### 2.2.2. Measurement Techniques

##### Scanning Electron Microscope

The morphological structure of BC was analyzed using two different Scanning Electron Microscopes (SEM): the Zeiss Ultra Plus Model SEM (Potsdam, Germany). The accelerating voltage was set to 2 kV.

##### Confocal Scanning IR Laser Microscope by LEXT-OLS Software

The surface roughness structure of the RTL-coated fabrics, both before and after coating, was analyzed using a Confocal Laser Scanning Microscope, Olympus Lext OLS3100 (OLYMPUS Corp., Tokyo, Japan) equipped with a 405 nm laser, LEXT-OLS Software version 5.0.9, following the ČSN EN ISO 21920-2 (014450) standard, [30].

##### Water Contact Angle

A droplet shape analyzer (DSA) equipped with ImageJ software version 1.54k, a OLYMPUS OM-D E-M5 Mark II (OLYMPUS Corp., Tokyo, Japan) camera, and OLYMPUS M.ZUIKO DIGITAL ED 60 mm F2.8 Macro Lens was used to deposit 0.05 mL droplets on the fabric surfaces to measure the hydrophobicity of dried samples through Water Contact Angle (WCA) tests. The surface characteristics were analyzed using ImageJ software.

##### Custom Built Dynamic Hot Plate Method

The method for testing the color change in the fabric involves using a heat control plate, with the temperature set at 37.5 °C. Test samples coated with thermochromic substances are cut to dimensions of 1.5 × 5.0 cm and attached to a prepared white cardboard sheet. This allows all test samples to be placed on the heat plate simultaneously. The color change behavior of each fabric type is observed and recorded using an OLYMPUS OM-D E-M5 Mark II camera over time.

**Figure 7:**
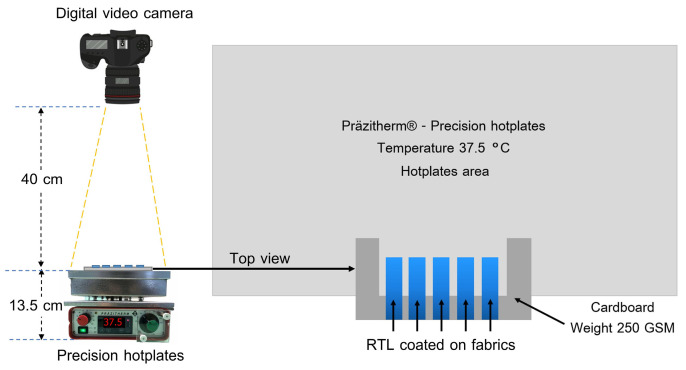
Schematic of the experimental setup for capturing video of the color-changing behavior.

The experiment used a precision hotplate from Präzitherm^®^ model PZ 28-1 (Harry Gestigkeit, Düsseldorf, Germany), Dimension hotplate 200 × 280 mm., the temperature preselection RT-110 °C, Hysteresis 0.1 ± K, power 500 W, Voltage 230 V, 50–60 Hz. The experiment involved placing RTL-coated fabric samples on a hot plate control system set to a temperature of 37.5 °C. As shown in Figure 7, the setup was divided into two distinct zones. The experimental zone contained fabric samples in direct contact with the hot plate, simulating an initial fever condition or an elevated body temperature of 37.5 °C. The second zone consisted of fabric adhered to a cardboard sheet 250 GSM, which acted as a heat-resistant barrier, preventing heat transfer and ensuring no color change in this area. This unaffected zone served as a reference to highlight changes observed in the experimental zone.

##### Thermal Properties

The Alambeta tester is a device designed to quickly assess both steady-state and transient thermal properties. It simulates the conditions of dry human skin by using mathematical analysis of the heat flow over time through the fabric being tested. The fabric is maintained at room temperature, while the measuring head, heated to 32 °C to mimic human skin temperature, uses an electrical heater. During testing, various fabrics are placed between the plates and compressed under a stress of 200 Pa. When the specimen is inserted, the measuring head lowers, contacts the fabric, and the heat flow data are processed by a computer to evaluate the thermo-physical properties of the sample [31,32,33,34]. The relationship between thermal conductivity (***λ***), fabric thickness (***h***), and thermal resistance (***γ***) is defined by Equation (1). This device partially replicates the heat transfer (q) Wm^−2^ from human skin to fabric during initial contact, under conditions without body movement or external airflow. Thermal resistance, expressed in m^2^KW^−1^, quantifies the fabric’s insulation capacity. The thermal resistance (***γ***) value is influenced by the fabric’s thermal conductivity (***λ***) [31,32].

(1) $$\gamma =\frac{h}{\lambda }$$

##### Water Vapor Permeability

The Permetest Skin Model (Sensora, Liberec, Czech Republic) was used to determine the relative water vapor permeability P wv (%), water vapor resistance Ret (m^2^ Pa/W). Before the measurement, the instrument was calibrated [35].

##### Antimicrobial Test

The antibacterial of textile materials was evaluated according to AATCC 147 [36]. The test was conducted using *Staphylococcus aureus* and *Klebsiella pneumoniae*. Fabric specimens were cut to appropriate sizes and placed on nutrient agar plates inoculated with bacterial cultures. The bacteria were streaked in parallel lines on the agar surface, and the fabric samples were carefully positioned over them. The plates were then incubated at 37 °C for 18–24 h to allow bacterial growth.

##### Thermal Image of Face Mask

To capture thermal images, a Hikvision DS-2TP31B-3AUF (Hangzhou Hikvision Digital Technology Co., Ltd., Hangzhou, China) handheld thermography camera was used. This device allowed for precise observation of temperature distribution across the face mask. A PVC head manikin was used as the test model. To simulate realistic thermal conditions, the manikin was modified with an electric USB-heated pad positioned around the cheek area. This setup enabled controlled temperature regulation, ensuring consistent heating for accurate observations.

##### Air Permeability of Face Mask

The air permeability test was conducted in accordance with the ISO 9237:1995 standard [37] using the Model M021A (SDL Atlas, Shanghai, China) air permeability tester. The test was performed with an air pressure differential of 100 Pa across the fabric surfaces over a test area of 20 cm^2^. Ready-to-use fabric mask products were tested, and their air permeability was measured in units of L/m^2^/s.

##### Durability Test of Face Mask

The durability test was conducted to evaluate the color fastness of the face maskto washing, following the ISO 105-C10:2006 (E) standard [38], Method A(1). The test was performed at 40 °C for 30 min using a soap solution containing 5 g/L of standard soap. After washing, the face mask was assessed for color change and color staining on six reference materials: acetate, cotton, nylon, polyester, acrylic, and wool. The results were rated based on standard evaluation criteria.

The water repellency test (spray test) was conducted in accordance with the ISO 4920:2012 (E) standard [39]. The test was performed after 10 cycles of water soaking, and the water repellency was evaluated using a rating scale from 0 to 5, where 0 complete wetting of entire face of the specimen and 5 represents no sticking or wetting of the specimen face. The results were recorded based on the standard assessment criteria.

## 3. Results and Discussion

### 3.1. Morphological and Microstructural Analysis of Fabrics

**Figure 8:**
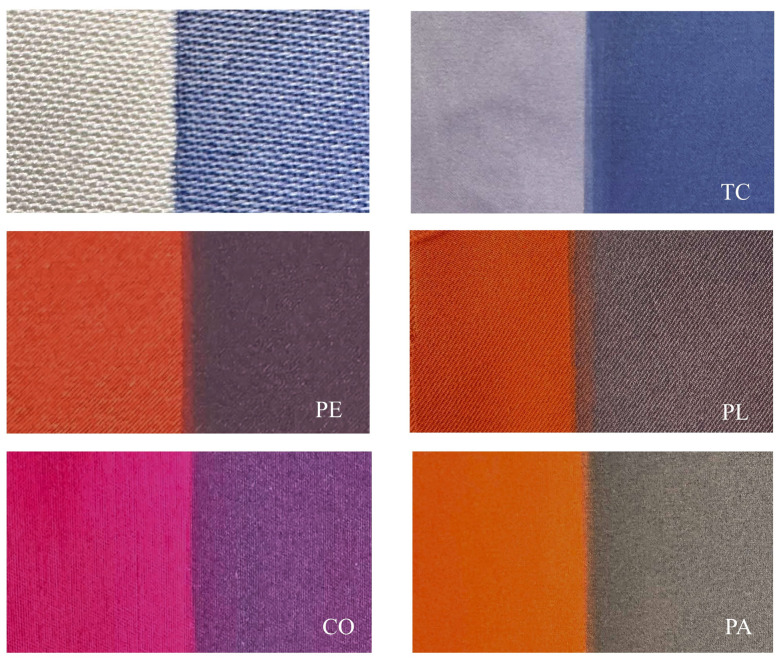
The comparative of fabric appearance with RTL.

The finished fabric appearance with RTL coating applied to various fabrics is displayed at the room temperature at approximately 25 °C. in Figure 8. The left side of each fabric sample shows the original color of the fabric, while the right side exhibits the color resulting from the application of the blue RTL coating. It is evident that the right side appears darker, particularly when compared to white fabric, which highlights the true color development. For the development of the fabric mask, original fabric colors in warm tones were selected, while the RTL coating was chosen in cool tones. This color psychology communicates temperature changes, warm tones indicate higher temperatures ≥37.5 °C, while cool tones appear when temperatures are <37.5 °C.

**Figure 9:**
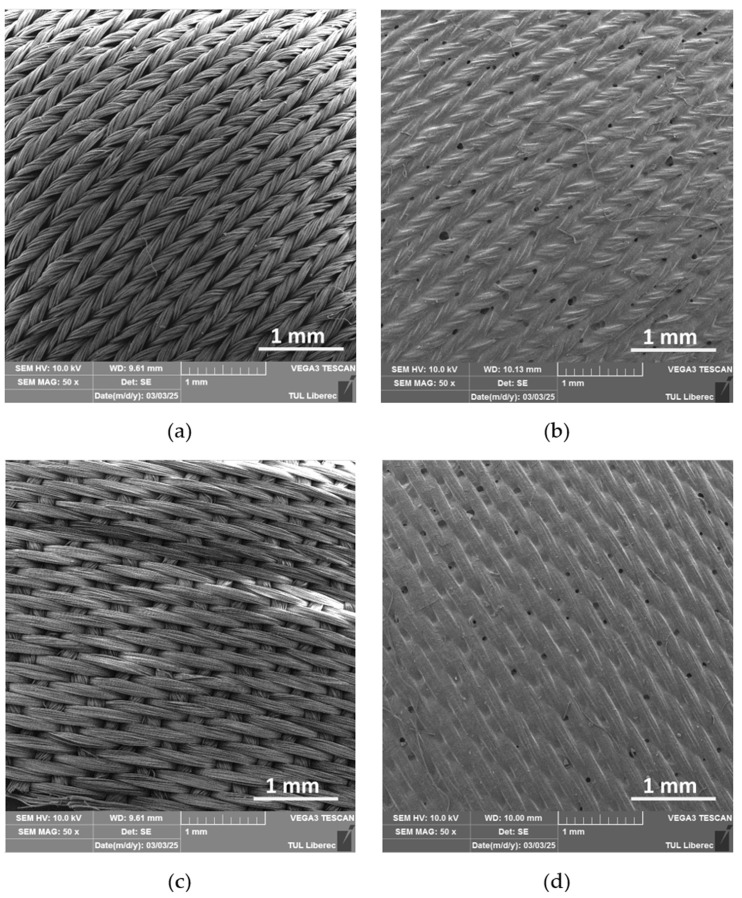
SEM surface image of the uncoated and coated RTL: (**a**) PA uncoated; (**b**) PA coated RTL; (**c**) PE uncoated; and (**d**) PE coated RTL.

A Scanning Electron Microscope (SEM) was used to analyze the surface morphology of selected fabric samples, PA and PE, before and after RTL coating, as shown in Figure 9a–d. The SEM images provided a detailed visual examination of the fabric’s surface structure. The analysis revealed that the uncoated fabric exhibited a natural fiber texture, while the coated fabric displayed a uniform and smooth RTL distribution. Additionally, the SEM images indicated that the coating adhered consistently, with minimal porosity.

**Figure 10:**
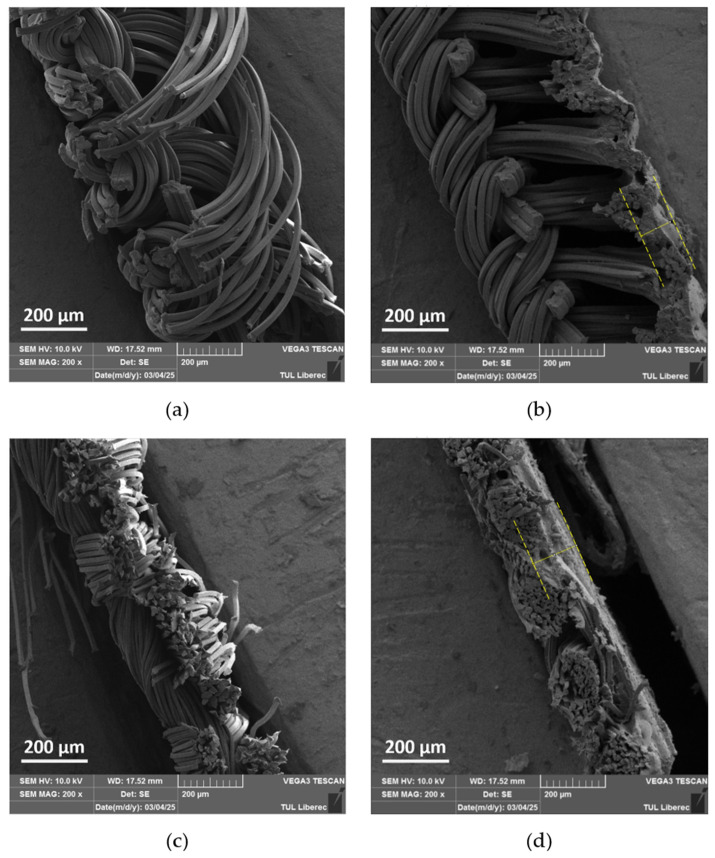
SEM cross-sectional image of the uncoated and coated. (**a**) PA uncoated; (**b**) PA coated RTL; (**c**) PE uncoated; and (**d**) PE coated RTL.

The SEM analysis examined the cross-sectional structure of uncoated and RTL-coated PA and PE fabrics, as shown in Figure 10a–d. Figure 10a illustrates the cross-section of PA fabric, which has a single jersey structure with a thickness of 0.65 mm. In contrast, Figure 10b shows the PA fabric coated with RTL, where the coating smooths the fabric surface and causes fibers to adhere more closely. The measured thickness of the RTL coating on the PA fabric surface at the yellow dash line is approximately 95.45 μm.

Figure 10c presents the cross-sectional structure of the PE fabric, revealing a thin satin structure with a thickness of 0.19 mm. In Figure 10d, the PE fabric exhibits significant RTL absorption within its structure, with the coating layer at the yellow dash line measuring approximately 143.83 μm. This enhanced absorption leads to a stronger interaction between the coating and the fabric structure. The differences in thickness and RTL absorption impact heat transfer, thereby influencing the color change behavior of RTL. The lower absorption of RTL in PA fabric may result in reduced thermal responsiveness and a less efficient color change, whereas the higher penetration of RTL in PE fabric enhances its thermal sensitivity.

### 3.2. Analysis of Surface Roughness

Table 4 displays images captured using an OLYMPUS LEXT OLS3100 confocal microscopy model for each fabric type. The first column, labeled “Uncoated”, shows the original fabric, revealing its color and structural characteristics. The second column presents fabrics coated with RTL using the screen printing process, demonstrating uniform application of the RTL coating and a darker shade resulting from the coating. The final column features magnified images of the RTL-coated fabric, providing a clear view of the RTL coating. It is evident that the coating fills the gaps between the fabric fibers, which reduces air permeability and contributes to an increase in fabric density.

### 3.3. Analysis of Surface Characteristics (Water Contact Angle)

**Figure 11:**
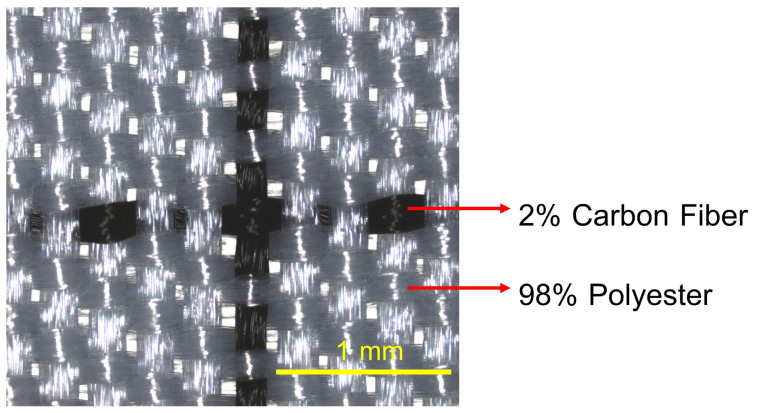
The confocal microscopy images of the face mask shell.

The outer shell of the face mask is made from a woven plain-weave fabric composed of 98% polyester microfiber blended with 2% micro carbon fibers, as shown in Figure 11. A detailed view of the fabric structure. The microscopy analysis clearly reveals the composition of the fabric, which consists of two distinct yarn types.

**Figure 12:**
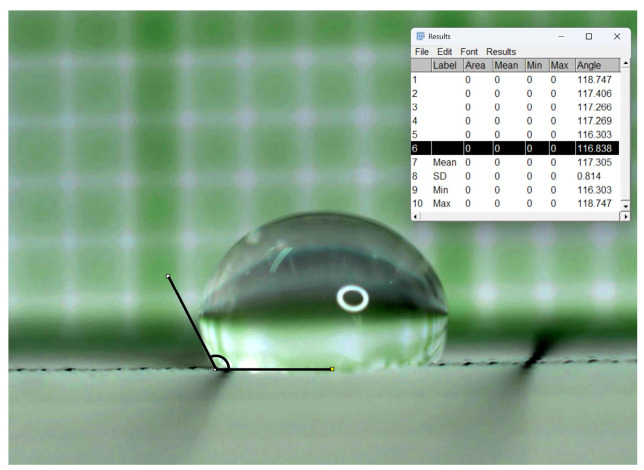
Analysis of surface characteristics WCA of shell fabric face mask.

The face mask shell was treated with antibacterial and water repellent and the surface characteristics were analyzed. A water droplet 0.5 mL was deposited on the hydrophobic surface, and the Water Contact Angle (WCA) was measured. The results indicated a WCA of approximately 116.84° at the time of 30th second, as shown in Figure 12.

**Figure 13:**
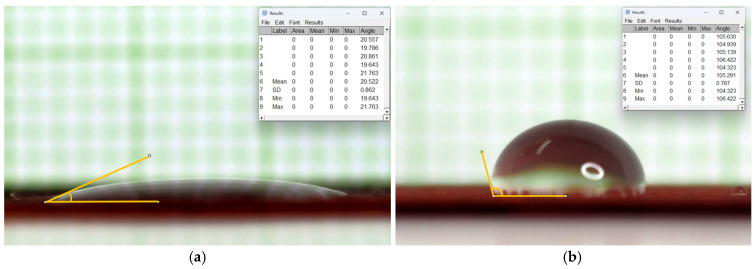
Analysis of surface characteristics WCA of PE fabric coated RTL. (**a**) Non-water-repellent treatment; (**b**) water-repellent treatment.

The measurement of the WCA on fabric coated with RTL revealed that when a 0.5 mL water droplet was placed on the PE fabric coated with RTL, the water was rapidly absorbed, as shown in Figure 13a. After 30 s, the droplet had completely penetrated the fabric, indicating significant water absorption. At this point, the WCA of the water droplet was measured at 20.52°, which led to an issue where the RTL-coated on the fabric changed color. As a result, RTL could not function effectively as a temperature-sensitive indicator, rendering the fabric mask ineffective when exposed to water or sweat.

To address this issue, a hydrophobic surface treatment was applied to the RTL-coated fabric of the sample PE. As shown in Figure 13b, the treated fabric exhibited improved hydrophobicity. After 30 s, the WCA of the water droplet on the treated fabric was measured at 105.29°, demonstrating significantly enhanced water repellent. The application of a hydrophobic surface treatment provides several benefits for the RTL-coated fabric in face masks. By preventing water absorption, it ensures that the RTL compound remains stable and effective as a temperature-sensitive indicator.

### 3.4. Analysis of the RTL in Relation to Time

**Figure 14:**
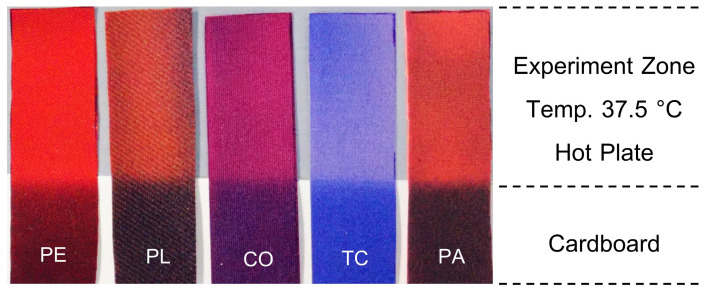
The appearance of color change in RTL fabric in 20 s.

The findings revealed that RTL-coated fabrics exhibited a noticeable color transition, taking over 20 s to complete across all fabric samples, as illustrated in Figure 14. However, the speed of the color change varied depending on the fabric type, as different materials displayed distinct levels of thermal conductivity and thermal absorptivity, which influenced their responsiveness to heat. Fabrics with higher thermal transfer properties facilitated quicker heat transfer, resulting in faster activation of the RTL coating, while less conductive fabrics demonstrated a slower response.

**Figure 15:**
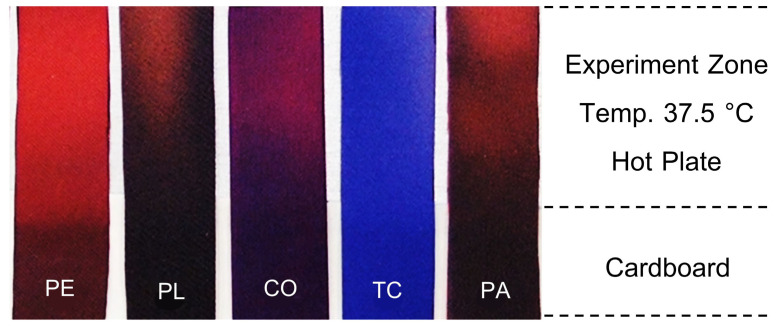
Range of color change from fixed temperature at the 4th second.

Figure 15 illustrates the behavior of RTL in different fabric types as their colors change, captured at the 4th second. The 100% Polyethylene (PE) fabric exhibited the quickest thermal response, with RTL-coated PE fabric showing improved thermal conductivity and absorptivity, enabling efficient heat distribution across the surface. As heat rapidly transfers through the fabric to the thermochromic material, the RTL coating temperature increases uniformly, leading to quicker and more consistent color transitions. These fabrics were highly responsive to thermal contact from the hot plate (simulating human skin) and reacted promptly with the RTL coating, resulting in rapid color changes. Conversely, fabrics with lower thermal conductivity experienced slower heat dissipation, causing uneven or localized color changes, as depicted in the corresponding figure.

**Figure 16:**
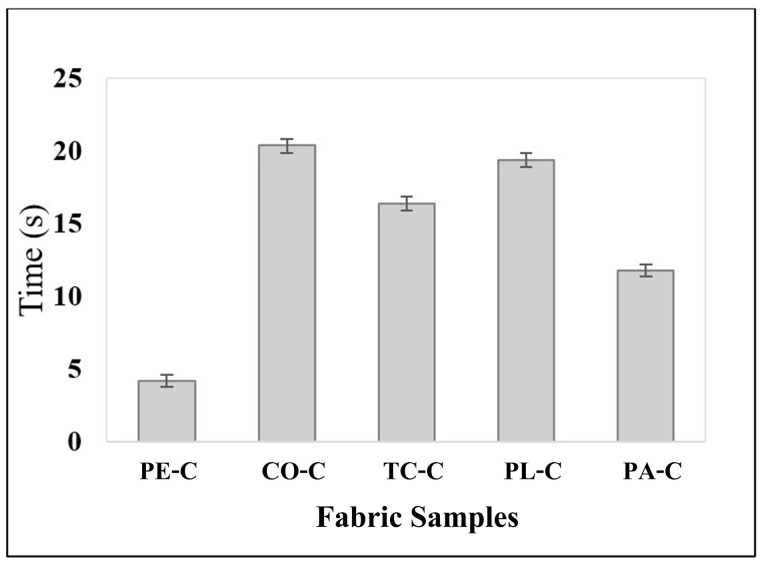
A graph comparing the color change duration of RTL.

The color-changing behavior of each fabric type coated with RTL was tested at a simulated temperature of 37.5 °C, during which the time required for a complete color transition across the fabric was observed and recorded. The results indicating the responsiveness and duration of the color change are presented in Table 5 and graphically illustrated in Figure 16.

The experimental findings indicate that the duration required for RTL color change varies significantly across different fabric types. Among the fabrics tested, PE exhibited the highest sensitivity to color change, with the shortest average response time of 4.20 (±0.392) seconds. This was followed by PA fabric, which demonstrated a comparatively faster response, achieving an average color change time of 11.80 (±0.392) seconds. The remaining fabric types TC, PL, and CO displayed progressively slower color change times, with recorded averages of 16.40 (±0.480) seconds, 19.40 (±0.480) seconds, and 20.40 (±0.480) seconds, respectively. These results highlight the distinct thermal responsiveness and efficiency of the RTL reaction across various fabrics.

These findings highlight the significance of fabric composition in RTL applications. The variation in response times suggests that selecting the appropriate fabric type is crucial for optimizing the performance of RTL textiles. For instance, fabrics with rapid thermal response may be more suitable for applications requiring quick temperature detection with real-time thermal feedback.

### 3.5. Analysis of Thermal Properties

In this phase, testing was conducted using the Alambeta tester to assess the thermal conductivity (***λ***), thermal resistance (***γ***), thermal absorptivity (***b***), and heat flow (***q***) of five fabric samples: PE, CO, TC, PL, and PA. Each fabric sample was evaluated under three conditions: the original uncoated fabric, the fabric coated with RTL, and the RTL-coated fabric layered with an additional inner lining made of cotton fabric (CO) no.3, as shown in Figure 6, to simulate the condition of wearing a fabric mask product (Coated + CO). The test results are summarized in Table 6.

**Figure 17:**
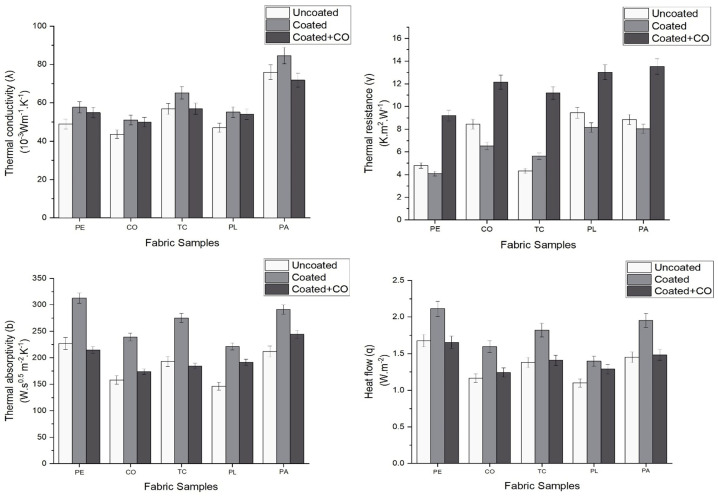
Graphs comparing the thermal properties of different types of fabrics and states.

From the graph shown in Figure 17, which compares fabrics under various conditions as previously described, it is evident that the PA fabric exhibits the highest thermal conductivity (***λ***) in the uncoated state at 75.95 (10^−3^ Wm^−1^K^−1^). In the RTL-coated state, the thermal conductivity value slightly increases due to the coating reducing the air gaps within the fabric structure on the surface, thereby enhancing the heat transfer efficiency. However, when an additional layer of cotton fabric is added (Coated + CO), the heat transfer efficiency decreases, as demonstrated by the test results.

The evaluation of thermal resistance (***γ***) demonstrates that fabric thickness is a critical factor influencing resistance to heat transfer. From the thermal resistance graph, it is evident that PA and PL fabrics exhibit higher thermal resistance compared to other fabrics, as described by the equation ***γ = h/λ***. The thickness of PA and PL fabrics, measuring 0.65 mm and 0.40 mm, respectively, is greater than that of other fabrics, resulting in their higher thermal resistance. However, when the fabrics are in the RTL-coated state, the thermal resistance slightly decreases due to the integration of the RTL coating, which enhances heat transfer efficiency. In the (Coated + CO) state, the addition of a cotton layer increases the overall fabric thickness, leading to a further rise in thermal resistance.

The test results for thermal absorptivity (***b***) and heat flow (***q***) demonstrate a clear correlation, as higher thermal absorptivity allows the fabric to absorb and distribute heat more efficiently, leading to an increase in heat flow. This indicates a positive relationship between thermal absorptivity and heat flow. The highest values were observed in RTL-coated PE fabric, with thermal absorptivity of 312.80 Ws^0.5^m^−2^K^−1^ and heat flow of 2.11 Wm^−2^, highlighting its superior thermal properties. Furthermore, the results for thermal absorptivity and heat flow align with the rate of color change in RTL-coated PE fabric, suggesting a link between thermal properties and the responsiveness of the RTL coating.

When evaluating fabrics for optimal heat transfer properties, relying solely on fiber type or fabric material based on thermal conductivity (***λ***) may not be sufficient. The fabric’s structure, particularly its thickness, significantly contributes to higher thermal resistance (***γ***). Experimental results indicate that thermal absorptivity (***b***) and heat flow (***q***) are more effective criteria for fabric selection, as they directly align with the performance observed in color-changing tests.

**Figure 18:**
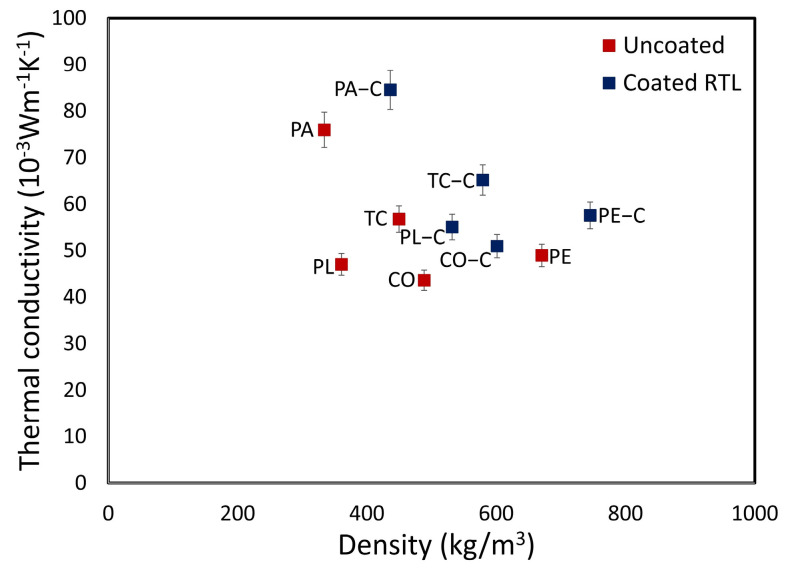
Thermal conductivity versus density plot comparing uncoated and RTL−coated fabrics.

Figure 18 clearly demonstrates that coating the fabric with RTL increases the density of each fabric type. Moreover, as the fabric density increases, thermal conductivity correspondingly rises. Based on the evaluation, PE fabric exhibits the highest thermal conductivity.

**Figure 19:**
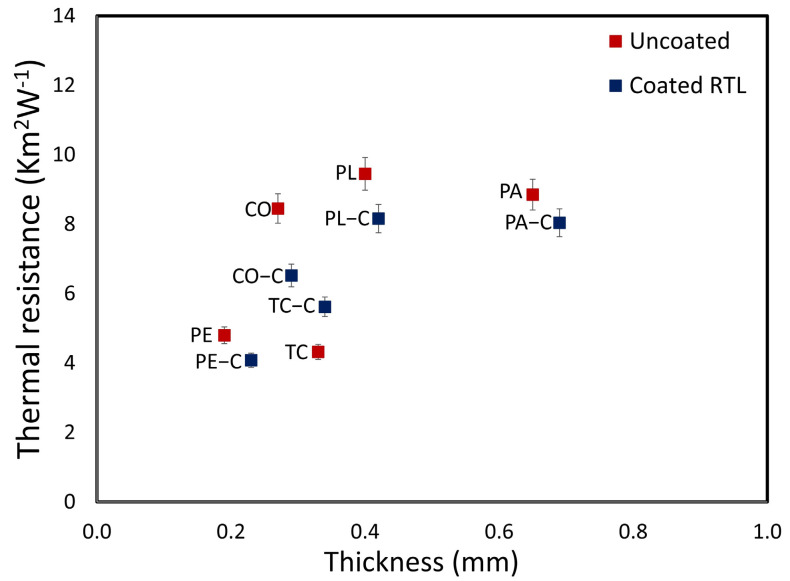
Thermal resistance versus thickness plot comparing uncoated and RTL−coated fabrics.

Figure 19 presents a graph of the relationship between thermal resistance and fabric thickness. It is observed that coating the fabric with RTL results in a slight increase in fabric thickness; however, it significantly reduces thermal resistance. This reduction occurs because the application of RTL enhances the adhesion and bonding of the fabric structure and fibers, leading to lower thermal resistance despite the increased fabric thickness.

**Figure 20:**
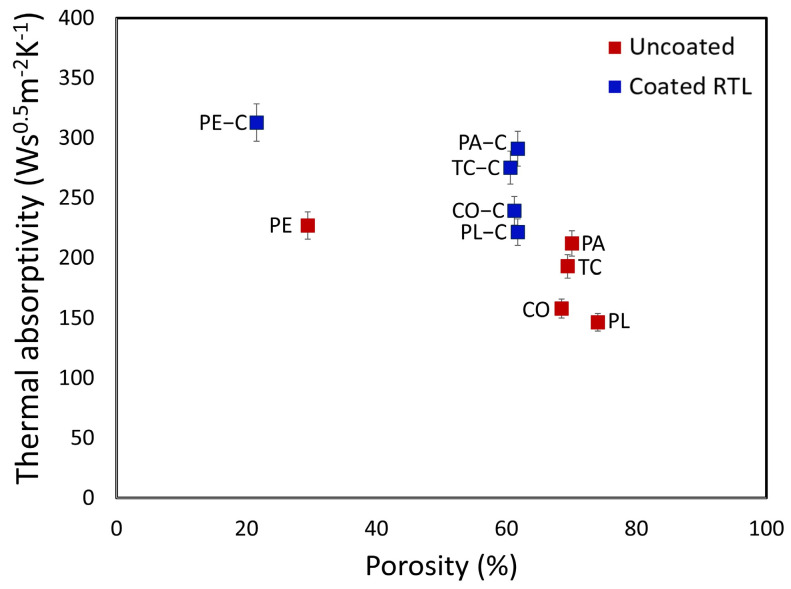
Thermal absorptivity versus porosity plot comparing uncoated and RTL-coated fabrics.

The graph of thermal absorptivity versus porosity illustrates that coating the fabric with RTL reduces the percentage of porosity while significantly increasing thermal absorptivity. This indicates that RTL enhances the fabric’s ability to absorb heat, making it more susceptible to color changes when exposed to high temperatures. Based on the analysis, PE−C fabric exhibits the highest thermal absorptivity, followed by PA−C fabric, as shown in Figure 20.

### 3.6. Water Vapor Permeability of the Fabric

The Permetest Skin Model (Sensora, Liberec, Czech Republic) was utilized to assess the percentage of Relative Water Vapor Permeability (RWVP) and Absolute Water Vapor Permeability (AWVP) of the tested fabric samples. The environmental conditions during testing included an inlet air temperature of approximately 24.63 °C and a relative humidity of 55.33%. The face mask consists of two layers, with the front breathable section at the center facilitating airflow. The outer shell fabric is a woven plain-weave textile composed of 98% polyester microfiber blended with 2% micro carbon fibers, while the inner lining is made of 100% cotton plain-weave fabric. The outer layer exhibited an RWVP of 60.15%, an AWVP of 3.9 Pa.m^2^.W^−1^, and a normalized heat flow of 79.55%. The inner layer showed an RWVP of 77.05%, an AWVP of 1.75 Pa.m^2^.W^−1^, and a normalized heat flow of 105.3%. These results indicate that the inner cotton layer has higher breathability and heat transfer efficiency compared to the outer polyester-carbon blend. This suggests that the inner layer plays a crucial role in moisture management and thermal comfort.

The results in Table 7 show a significant drop in RWVP after coating, with coated fabrics exhibiting much lower permeability. Some fabrics, like PE and PA, were completely impermeable, yielding no initial measurements. For measurable coated samples, testing exceeded seven minutes, indicating extremely low permeability. Coatings typically reduce breathability by acting as a moisture barrier, aligning with prior research on their restrictive effect on vapor transmission. In the specific case of the RTL-coated face mask, the placement of the coating was strategically chosen to enhance moisture resistance in areas that come into direct contact with the cheeks. Since these areas do not require high breathability, the coating improves functionality by preventing moisture accumulation while ensuring the fabric’s temperature indicator properties remain effective.

**Figure 21:**
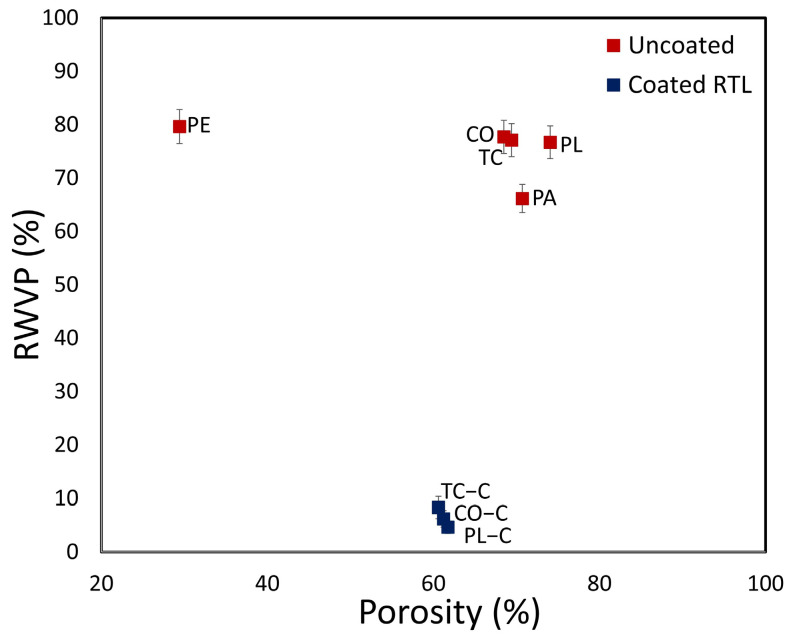
RWVP versus porosity plot comparing uncoated and RTL-coated fabrics.

The graph in Figure 21 illustrates %RWVP versus %porosity, and demonstrates that coating the fabric with RTL reduces the porosity percentage. Moreover, it is evident that relative water vapor permeability (%RWVP) is inversely correlated with porosity percentage. Notably, in the RTL-coated fabric, the %RWVP significantly decreases due to the RTL coating, which acts as a barrier to water vapor transmission. However, since the RTL-coated fabric is specifically designed to be positioned in close contact with the cheek area, it does not affect breathability. Additionally, it helps reduce moisture retention in the fabric, thereby enhancing the overall effectiveness of the RTL coating.

### 3.7. Analysis of Antibacterial and Water-Repellent Performance of Fabric

**Figure 22:**
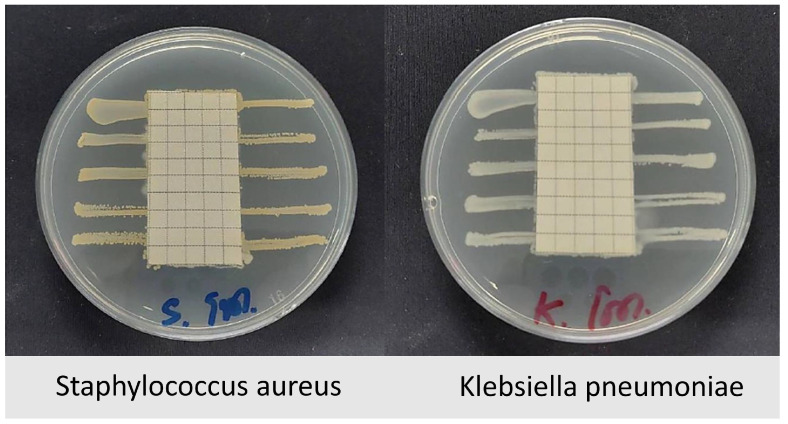
The results antimicrobial fabric tests standard AATCC147.

The antibacterial and water-repellent performance of treated fabric face masks was evaluated through standardized testing protocols. An antimicrobial assessment conducted according to AATCC 147 revealed contact-based antibacterial activity against both *Staphylococcus aureus* and *Klebsiella pneumoniae* [36]. While no inhibition zone was observed (Figure 22), the complete suppression of bacterial growth beneath the fabric specimen indicates effective localized antimicrobial action. This phenomenon suggests the presence of non-leaching antimicrobial agents that function through direct contact mechanisms rather than diffusible compounds. Such contact-active properties are particularly advantageous for medical and hygienic textile applications where preventing surface microbial colonization is critical. Additionally, the water repellency test (spray test) conducted in accordance with ISO 4920:2012(E) [39] showed that the fabric maintained a water repellency rating of 4 (indicating slight random sticking or wetting of the specimen surface) even after 10 cycles of water soaking. This rating met the acceptable standard of ≥3 for all tested materials. Furthermore, the formaldehyde content analysis, based on ISO 14184-1:2011 standards [40], indicated that the formaldehyde content of the fabric shell was 15 mg/kg, which is well below the standard textile limit of 75 mg/kg.

### 3.8. Analysis of Thermal Imaging of Face Mask

**Figure 23:**
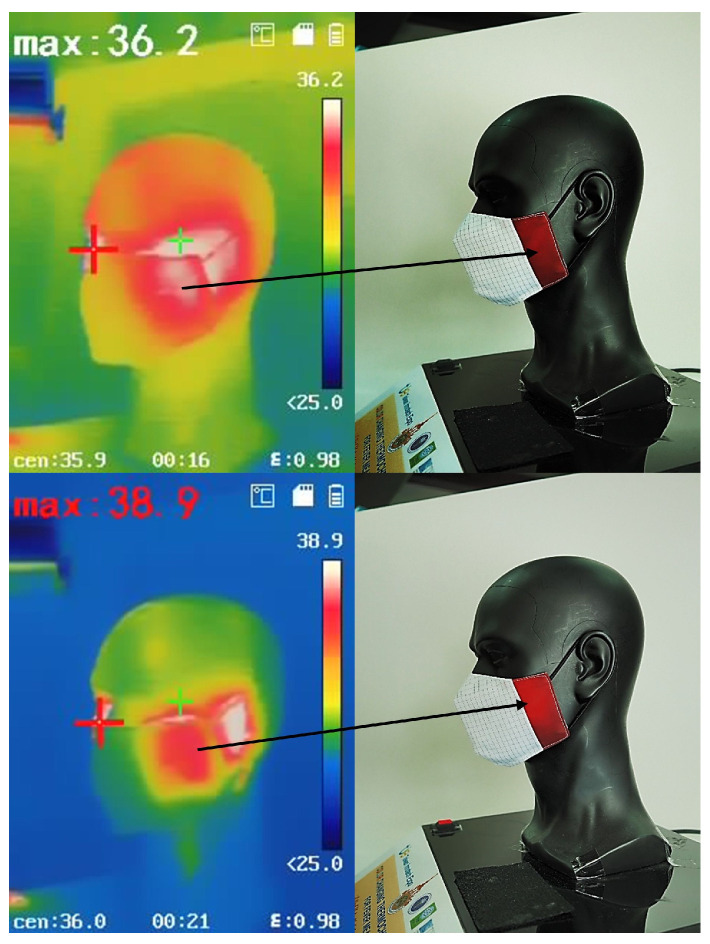
Thermal image analysis of color changes in the face mask at different temperatures.

The thermal imaging process focused on monitoring the color changes in the face mask as a response to temperature variations. Images were captured at different temperatures, with the maximum recorded temperatures being 36.2 °C and 38.9 °C. The comparison of these images allowed for an assessment of the temperature indicator functionality of the PE-coated RTL sample. As shown in Figure 23, the face mask exhibited a noticeable color change when the temperature exceeded 37.5 °C. This confirms that the temperature-sensitive coating effectively responds to heat variations, providing a visual indication of temperature fluctuations. The distinct color transition observed in the thermal images highlights the potential of the coated face mask for real-time temperature monitoring.

The results demonstrate that the PE-coated RTL sample successfully functions as a temperature indicator. The clear color change at temperatures above 37.5 °C suggests that the coating composition is well-calibrated to detect critical temperature thresholds. This property is particularly useful for applications where visual temperature cues are essential, such as health monitoring and personal protective equipment.

**Figure 24:**
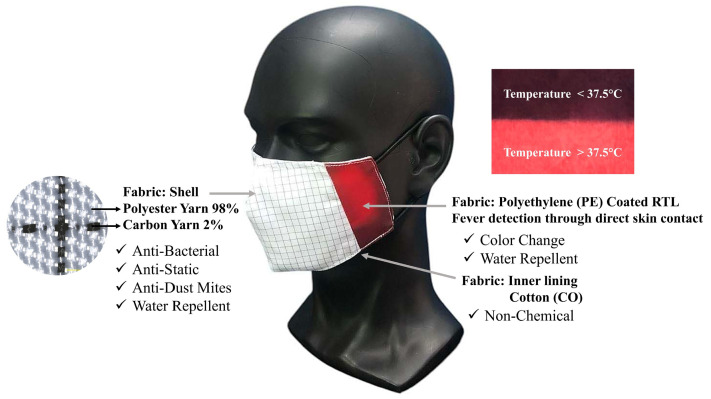
The functionality of the face mask for fever detection applications.

The ability of the face mask to change color in response to temperature variations aligns with previous studies on thermochromic materials of RTL. The observed changes indicate that the coated fabric can serve as an effective real-time temperature indicator. Moreover, the functionality of the face mask for fever detection applications in practical applications and further development is illustrated in Figure 24. These results reinforce the potential of RTL coatings for use in health monitoring and protective wear.

The air permeability test for the face mask was conducted in accordance with the ISO 9237:1995 standard [37], using an air pressure of 100 Pa over a 20 cm^2^ test area. The measured air permeability of the fabric face mask was 107.31 L/m^2^/s, exceeding the required minimum of 96 L/m^2^/s as specified by Smart Fabric face mask. This result confirms that the tested fabric meets the acceptable standard for fabric face masks in terms of breathability.

### 3.9. Durability of the Face Mask

The durability test was conducted to assess the color fastness of the face mask fabric to washing, following the ISO 105-C10:2006 (E) standard, Method A(1) [38]. The test evaluated color change and color staining on six reference materials: acetate, cotton, nylon, polyester, acrylic, and wool. The results met the acceptable standard of ≥3 for all tested materials, as presented in Table 8.

The water repellency test (spray test) was conducted in accordance with the ISO 4920:2012 (E) standard [39]. After 10 cycles of water soaking, the fabric received a rating of 4, indicating slight random sticking or wetting of the specimen. This result meets the acceptable requirement of the standard, which specifies that the fabric should have a rating of ≥3.

## 4. Conclusions

The conclusion of this research was the development of a color-changing fabric face mask for fever detection applications. The study focused on selecting fabrics coated with RTL, a melting point temperature of 37.5 °C. Testing was conducted to identify fabrics with rapid color-changing capabilities by analyzing their thermal properties. It was concluded that fabrics with higher thermal conductivity (***λ***), thermal absorptivity (***b***), and heat flow (q) exhibited better color change responsiveness. PE fabric demonstrated a thermal absorptivity of ***b*** = 312.8 Ws^0.5^m^−2^K^−1^ and heat flow of ***q*** = 2.11 Wm^−2^, higher than other fabrics, enabling a rapid color change reaction time of approximately 4.20 s. When evaluating fabrics for optimal heat transfer properties, relying solely on fiber type or fabric material based on thermal conductivity (***λ***) may be insufficient. The fabric’s structure, particularly its thickness, plays a crucial role in increasing thermal resistance (***γ***). Experimental findings suggest that thermal absorptivity (***b***) and heat flow (***q***) are more reliable criteria for fabric selection, as they directly correlate with performance in color-changing tests.

In addition to selecting RTL-coated fabrics with rapid color-changing capabilities, the design and performance enhancement of the fabric face mask were also emphasized. This included pattern creation using the draping technique, selecting outer shell fabrics with anti-static and dust mite protection properties, and incorporating additional functionalities such as antibacterial resistance and water repellency. These efforts ensure the effectiveness of the color-changing fabric face mask for fever detection applications. However, the color-changing efficiency of the RTL facemask is influenced by ambient temperature, with both high and low extremes affecting its performance. For optimal results, the mask should be used in moderate indoor environments such as hospitals, schools, airports, and industrial workplaces. Our future research will focus on the wearer trials under different conditions for subjective face mask comfort evaluation. User feedback and standardized comfort rating scales should evaluate factors such as breathability, thermal sensation, skin irritation, and overall fit. Research should examine how extended mask usage affects both user satisfaction and adherence to wearing guidelines. A thorough examination of these factors will yield essential insights that help improve mask design to boost comfort yet maintain protective standards.

## Figures and Tables

**Table 1:** Physical properties of uncoated fabrics.

Sample	Fabric	Symbol	Structure	Thickness (mm)	Weight (g/m^2^)	Density (kg/m^3^)	Porosity (%)
1	Polyethylene	PE	Satin weave	0.19	127.39	670.47	29.42
2	Cotton	CO	Pain weave	0.27	131.90	488.52	68.48
3	Cotton 50% and Polyester 50%	TC	Pain weave	0.33	148.38	449.64	69.41
4	Polyester	PL	Twill weave	0.40	144.25	360.63	74.06
5	Polyamide	PA	Single jersey	0.65	217.14	334.06	70.70

**Table 2:** Recipe of the pigment printing paste.

Constituent	Weight Percent (wt%)
Binder	10
Thickener	8
Emulsifier	2
Oil	4
Water	76

**Table 3:** Physical properties of coated fabrics.

Sample	Symbol	Structure	Thickness (mm)	Weight (g/m^2^)	Density (kg/m^3^)	Porosity (%)
1	PE−C	Satin weave	0.23	171.42	745.30	21.55
2	CO−C	Pain weave	0.29	174.35	601.21	61.21
3	TC−C	Pain weave	0.34	196.91	579.15	60.60
4	PL−C	Twill weave	0.42	223.35	531.79	61.74
5	PA−C	Single jersey	0.69	300.94	436.14	61.74

**Table 4:** The confocal microscopy images before and after fabrics coated with RTL.

Fabric	Uncoated (Scale Bar: 1 mm)	Coated RTL (Scale Bar: 1 mm)	Coated RTL (Scale Bar: 200 µm)
PE	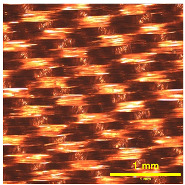	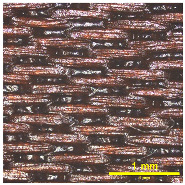	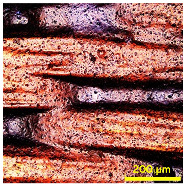
CO	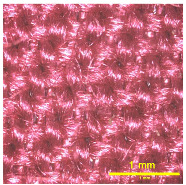	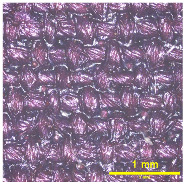	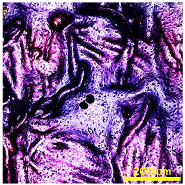
TC	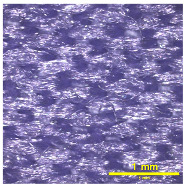	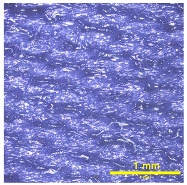	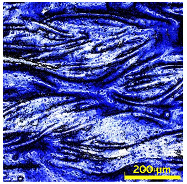
PL	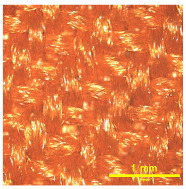	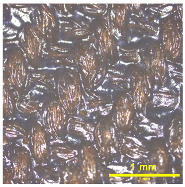	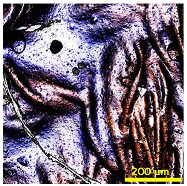
PA	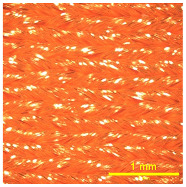	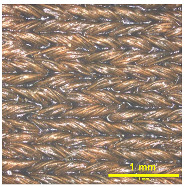	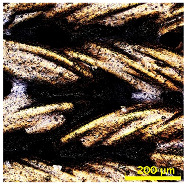

**Table 5:** The rate of color change sensitivity of fabrics coated with RTL.

Symbol	PE−C	CO−C	TC−C	PL−C	PA−C
Time (s)	4.20 (±0.392)	20.40 (±0.480)	16.40 (±0.480)	19.40 (±0.480)	11.80 (±0.392)
SD	0.447	0.548	0.548	0.548	0.447
CV%	10.648	2.685	3.340	2.823	3.790

**Table 6:** The results of the thermal properties of the fabric types under three different conditions.

**Symbol**	**Thermal Conductivity (λ)** **(10^−3^Wm^−1^K^−1^)**			**Thermal Resistance (γ)** **(Km^2^W^−1^)**		
	**Uncoated**	**Coated**	**Coated + CO**	**Uncoated**	**Coated**	**Coated + CO**
PE	48.95	57.60	54.83	4.80	4.08	9.20
CO	43.60	50.96	49.88	8.45	6.52	12.15
TC	56.80	65.20	56.93	4.32	5.62	11.20
PL	47.00	55.10	53.98	9.45	8.16	13.02
PA	75.95	84.56	71.85	8.85	8.04	13.53
**Symbol**	**Thermal Absorptivity (b)** **(Ws^0.5^m^−2^K^−1^)**			**Heat Flow (** q **)** **(Wm^−2^)**		
	**Uncoated**	**Coated**	**Coated + CO**	**Uncoated**	**Coated**	**Coated + CO**
PE	227.00	312.80	214.75	1.68	2.11	1.66
CO	158.00	239.20	173.75	1.17	1.60	1.24
TC	193.00	275.20	184.25	1.38	1.82	1.41
PL	146.50	221.40	191.20	1.10	1.40	1.29
PA	212.00	291.00	244.50	1.45	1.95	1.48

**Table 7:** Water vapor permeability of fabric uncoated and coated RTL.

Symbol	Uncoated		
	RWVP (%)	AWVP (Pa.m^2^.W^−1^)	Normalized Heat Flow (%)
PE	48.95	57.60	54.83
CO	43.60	50.96	49.88
TC	56.80	65.20	56.93
PL	47.00	55.10	53.98
PA	75.95	84.56	71.85
	**Coated RTL**		
PE-C	-	-	-
CO-C	6.2	88.9	8.6
TC-C	8.3	64.6	11.6
PL-C	4.65	123.05	6.15
PA-C	-	-	-

**Table 8:** Results of color fastness to washing for the face mask according to ISO 105-C10:2006 (E) standard.

Color Fastness	Smart Fabric’s Requirement	Rating
Color change	≥3	4
Color staining	≥3	
- Acetate		3–4
- Cotton		4
- Nylon		4
- Polyester		4
- Acrylic		4–5
- Wool		4–5

Remark(s): Rating 5 = No color change/staining, 4 = Slightly color change/staining, 3 = Noticeable color change/staining, 2 = Considerable color change/staining, and 1 = Excessive color change/staining.

## Data Availability

The original contributions presented in this study are included in the article. Further inquiries can be directed to the corresponding authors.
